# Estimating the burden of antimicrobial resistance in Malawi: protocol for a prospective observational study of the morbidity, mortality and economic cost of third-generation cephalosporin resistant bloodstream infection

**DOI:** 10.12688/wellcomeopenres.15719.2

**Published:** 2020-06-01

**Authors:** Rebecca Lester, Hendran Maheswaran, Christopher P. Jewell, David G. Lalloo, Nicholas A. Feasey

**Affiliations:** 1 Liverpool School of Tropical Medicine, Liverpool, UK; 2 Malawi Liverpool Wellcome Trust Clinical Research Programme, Blantyre, Malawi; 3 Institute of Population Health Sciences, University of Liverpool, Liverpool, UK; 4 Centre for Health Informatics, Computing and Statistics, Lancaster University, Lancaster, UK

**Keywords:** Enterobacterales, Extended-spectrum beta-lactamase, Third-generation cephalosporin, Africa south of the Sahara, Antimicrobial resistance

## Abstract

**Introduction: **Antimicrobial resistance (AMR) is a global public health concern, but the problems are context specific, with each county or setting facing differing challenges. In sub-Saharan Africa, third-generation cephalosporin resistant Enterobacterales (3GCR-E) are of particular concern, given the widespread reliance on ceftriaxone for treatment of severe infection in this setting. In Malawi, despite rising prevalence of 3GCR-E, the health-impact of these infections has not been described. This study is designed to estimate attributable mortality, morbidity and economic cost of 3GCR-E bloodstream infection (BSI) in a large, urban hospital.

**Methods: **This study will investigate the burden of AMR by recruiting a a prospective longitudinal cohort of patients who have bloodstream infection with 3GCR-E, at Queen Elizabeth Central Hospital, Blantyre, Malawi. Patients whose blood culture is positive for either third-generation cephalosporin susceptible (3GC-S) or third-generation resistant (3GC-R) Enterobacterales will be enrolled and provide clinical and healthcare economic data. Patients will be followed throughout their hospital stay and to 6-months post discharge. The primary outcomes for the study are mortality and morbidity from 3GCR-E. Healthcare economic outcomes will be assessed by comparing healthcare provider costs, indirect patient costs and health-related quality of life outcomes in patients with 3GC-S and 3GC-R BSI. Based on our observation that some patients with clinical suspicion of sepsis and 3GC-R BSI are surviving without an effective antibiotic, we review each patient prospectively and classify what role the isolated bacteria is playing in the patient’s clinical presentation. Each BSI episode will be classified into the following categories: definite Gram-negative sepsis, probable Gram-negative sepsis, transient or occult bacteraemia, or contaminated blood culture. These classifications will be incorporated into our analysis.

**Ethics and dissemination: **The study protocol has been approved by the Malawi College of Medicine Research Ethics Committee and by the Liverpool School of Tropical Medicine Research Ethics committee.

## Introduction

### Antimicrobial resistance in sub-Saharan Africa

Antimicrobial resistance (AMR) is a property of micro-organisms which have evolved to survive exposure to the antimicrobials previously successfully used to treat them. Drug-resistant infections (DRIs) occur when AMR bacteria cause infection and have become a global public health problem ^1^. In high-income countries, DRIs frequently remain amenable to therapy, albeit with more expensive antibiotics, thus incurring increase in healthcare costs. The greatest burden of DRIs, however, is expected to occur in low and middle-income countries, where alternative antibiotics are frequently unavailable or prohibitively expensive, and the morbidity and mortality from these infections is predicted to be high ^2^.

Third-generation cephalosporin resistant Enterobacterales (3GCR-E), have been identified by the World Health Organization (WHO) as critical priority pathogens on which national AMR programmes should focus their surveillance and reporting ^2^. These pathogens are of particular importance in sub-Saharan African hospitals, where the third-generation cephalosporin, ceftriaxone, is frequently relied upon in the empirical treatment of sepsis ^3– 5^.

Median proportions of third-generation cephalosporin resistance amongst bloodstream Enterobacterales in sub-Saharan Africa (sSA) are high, approaching 15% in *Escherichia coli* and 50% in *Klebsiella* ^6^. The most comprehensive published information on AMR trends in sSA, comes from Malawi, where blood culture surveillance data from patients presenting to Queen Elizabeth Central Hospital (QECH), has shown a recent, rapid rise in third-generation cephalosporin resistance amongst Enterobacterales ^7^. Between 2003 and 2016, 3GC resistance rose from 0·7% to 30·3% in *E.coli* and from 11·8% to 90·5% in *Klebsiella*, contemporaneous to the widespread roll-out of ceftriaxone in the hospital since 2005 ^7^. The lack of availability and prohibitive expense of alternatives to ceftriaxone, means that unlike in high income countries, these infections are locally untreatable.

### Knowledge gaps

Despite the rising prevalence of third-generation cephalosporin resistance amongst key pathogens and a reliance on ceftriaxone for management of infection, the health impact of 3GC-R BSI in sSA has not been described ^6^. Findings from large-scale cohorts in high-income settings, suggest that these infections are associated with adverse patient outcomes, including high mortality, length of hospital stay and total healthcare costs ^8– 11^, but only one published study from sSA has investigated health burden from AMR, finding a significant impact of third-generation cephalosporin resistance on mortality ^12^. Malawi is one of the few countries in sSA with a long-term blood-culture service, but the Malawian dataset, though comprehensive in its AMR prevalence and incidence estimates, does not link drug-resistant infections to clinical metadata such as patient outcomes ^7^.

### Study approach

This study is designed to help address these knowledge gaps and aims to estimate the attributable mortality, morbidity and economic cost of 3GC-R BSI infection in Malawi by recruiting a prospective longitudinal cohort of patients who have bloodstream infection (BSI) with Enterobacterales. The burden and presentation of clinical infectious disease in sSA may not be the same as in resource rich settings, therefore a key strength of this study is its prospective nature, which enables investigators to collect high quality data by reviewing every patient alive by the time their culture is positive and to determine the role the isolated pathogen is playing in the clinical presentation.

The methodological challenges involved in designing studies that aim to accurately estimate the burden of AMR on patients and health systems, have recently been debated ^13^. This consortium reflected upon the need to use the counterfactual approach to assessing burden of AMR, assuming that the likelihood of death would have been different if the pathogen had been susceptible. The counterfactual assumption is that death would not have occurred if the organism causing the infection had been drug-susceptible (or if there had been no infection) ^14, 15^. This assumption allows for comparison between patients with resistant and susceptible bacterial infection ^13^. This counterfactual approach is the one taken by this study, in which attributable mortality and other health outcomes will be estimated by making comparisons between patients with 3GC-R and comparable 3GC-S bloodstream infections, recruited in a prospective observational cohort.

Typically, a blood culture yielding a member of the family Enterobacterales would be considered to be of high clinical significance and thus trigger antimicrobial therapy to be commenced or refined. It would be unusual to classify such an organism as a contaminant ^16^. Consequently, Enterobacterales are routinely included in AMR surveillance studies without further consideration ^11^. In the Malawian context, however, the limited availability of carbapenems and aminoglycosides means that patients whose blood culture is positive for 3GCR-E frequently remain untreated with an agent to which the isolate is susceptible. Despite this, patients often recover, posing the question, what role is the bacterial isolate playing in the patient’s presentation?

If Enterobacterales are genuinely present in blood cultures as contaminants or cryptic organisms in significant numbers, it would have profound implications for burden of AMR studies. Before the disease burden attributable to resistant bloodstream infections can be estimated, the role a given blood culture isolate is playing in the clinical episode must be characterised. This protocol has therefore been designed to leverage the prospective nature of this study to propose a method for classifying the impact of each positive blood culture on a patient, and the subsequent incorporation of these classifications into our analysis of morbidity, mortality and cost. We further describe the clinical, laboratory, economic, data-management and ethical components of the study.

## Methods

### Study design

The study is a prospective longitudinal observational cohort of patients whose blood culture is positive for Gram-negative pathogens, excluding Salmonellae, regardless of sensitivity pattern. Patients whose blood culture isolate is susceptible to ceftriaxone will be recruited following the same procedures as those whose blood culture isolate is resistant, so that mortality, morbidity and economic comparisons can be made between the two groups. Detailed inclusion and exclusion criteria are shown in Table 1. Salmonellae will be excluded as 3GC-R in non-Typhoidal *Salmonella* remains sporadic and has not yet been reported in *Salmonella* Typhi in Malawi.

**Table 1.** Study inclusion and exclusion criteria.

Inclusion criteria
Blood culture is positive for non- *Salmonella* Enterobacterales or Acinetobacter ^*^
Patient is an inpatient at QECH or can be contacted for admission or assessment
Exclusion criteria
Blood culture is positive for *Salmonella enterica* (any serovariant)
Patient is unable to provide informed consent and there is no representative to provide informed consent
Patient speaks neither English or Chichewa

*During the set-up period for the study, it became clear that 3GC-R *Acinetobacter* spp. were an emerging problem at QECH, particularly amongst neonates. Acinetobacter are closely related to *Enterobacterales*, often sharing similar AMR profiles, and given their importance as a nosocomial pathogen, these patients will be included. However for analysis purposes we may do the primary analysis on the whole cohort and then Enterobacterales alone

### Study site

Malawi has a population of 17.5 million people and is classified as low income by the World Bank (2018 GDP of US$ 7.1 Billion, ranking 149 ^th^ out of 205 economies) ^17^. Blantyre is the second city of Malawi, with a population of 800,264 and is located in Blantyre district, population 995,000 (2018 census) Life expectancy at birth is estimated at 64.3 years ^18^. Malawi was one of 10 low-income countries to reduce its under-five mortality by at least two-thirds between 1990–2018, but infant and neonatal mortality remain high, at 38 and 22 per 1000 live births ^19^. The study will be being conducted at Queen Elizabeth Central Hospital (QECH), the largest government hospital in the country. QECH provides free healthcare to Blantyre and the surrounding districts, plus tertiary care to Malawi’s Southern region. It receives approximately 10,000 adult and 30,000 paediatric admissions per year ^8^ and has 1,300 beds, frequently operating above capacity. In July 2017, the Mercy James Centre (MJC) for Paediatric Surgery and Intensive care was opened as a separate 50-bedded building, operating as part of QECH. MJC receives approximately 1,600 admissions per year and houses the country’s only Paediatric Intensive Care Unit (PICU).

### Blood culture service

A diagnostic blood-culture service, provided through the Malawi-Liverpool Wellcome Trust Clinical Research Programme (MLW) was established in 1998. MLW is affiliated with the Malawi College of Medicine and operates this service, 7 days/week, providing free aerobic blood cultures and cerebral spinal fluid (CSF) analysis to adult medical and paediatric patients. From March 2018, this service was extended to the Department of Obstetrics and Gynaecology, with a limited number of blood cultures offered per month.

Clinical blood culture protocols at QECH state that in adults, 7–10mls of blood should be taken in patients presenting to the emergency department with a fever (axillary temperature > 37.5C) or clinical suspicion of sepsis, severe sepsis or septic shock. In children, 1–2 mls of blood is taken in patients with non-focal febrile illness and a negative malaria test or in children with malaria whose fever persists despite treatment. A blood culture is also recommended in all premature or febrile neonates who are admitted to the neonatal unit. In a busy hospital with constrained resources and limited alternative diagnostics, blood cultures are often done on patients who do not fulfil these criteria, but at the discretion of the attending clinician. These patients will not be excluded from our analysis. The volume of blood taken for cultures in children in this setting is often low, potentially introducing bias to the study and selecting for patients with high bacterial load. This will be noted as a potential limitation to the study since resources for routinely weighing culture bottles are not available.

In the MLW laboratory, blood is inoculated into a single aerobic bottle using the automated BacT/ALERT system (bioMerieux, France). Enterobacterales and Acinetobacter are identified to species level using Analytical Profile Index testing (API) (bioMerieux, France). Before March 2019, antimicrobial sensitivity testing (AST) was carried out as per British Society of Antimicrobial Chemotherapy (BSAC) guidelines ^20^, and from March 2019, as per European Committee on Antimicrobial Susceptibility Testing (EUCAST) guidelines ^21^ ( Table 2). All blood culture isolates for patients recruited to the study will be regrown by a study laboratory technician and AST carried out as per EUCAST. The direct colony suspension method will be used to make a suspension of pure colony in 1ml 0.9% sterile saline solution, to the density of 0.5 McFarland turbidity standard. The resulting suspension will be streaked evenly onto Muller-Hinton agar (MHA), aiming for confluent bacteria growth. Antimicrobial discs will be applied and the resulting AST plates incubated at 35°C for 18 (+/-2 hours). Zones of inhibition for each antimicrobial will be measured to the nearest millimeter and susceptibility categories interpreted according to EUCAST breakpoint tables ^22^. The laboratory adheres to UK National External Quality Assessment Service (NEQAS) ^23^.

**Table 2.** Antimicrobial discs used in AST for blood culture isolates.

Enterobacterales	*Acinetobacter*
Co-trimoxazole 25 µg (SXT25)	Co-trimoxazole 25 µg (SXT25)
Gentamicin 10 µg (CN10)	Gentamicin 10 µg (CN10)
Ciprofloxacin 5 µg (CIP5)	Ciprofloxacin 5 µg (CIP5)
Meropenem 10 µg (MEM10)	Meropenem 10 µg (MEM10)
Amikacin 30 µg (AK30)	Amikacin 30 µg (AK30)
Chloramphenicol 30 µg (C30)	
Piperacilin-tazobactam (PTZ 36)	
Co-amoxiclav (AUG 30)	
Cefpodoxime (CPD10)	
Cefoxitin (FOX 30)	
Ceftriaxone (CRO30)	

For any Enterobacterales resistant to one or both of cefpodoxime or ceftriaxone on AST, extended spectrum beta-lactamase (ESBL) production will be confirmed using the species dependent combination disc method. The isolate will be cultured overnight on MHA with discs of cefotaxime and ceftazidime (30 micrograms) with and without clavulanic acid (10 micrograms). For all organisms capable of carrying chromosomal AmpC ( *Enterobacter* spp., *Serratia marcesens*., *Citrobacter freundii*, *Providencia stuartii*, *Morganella morganii*, *Hafnia alvei*), an AmpC-stable cephalosporin, cefipime (30 micrograms), will be used with and without clavulanic acid (10 micrograms). ESBL production is confirmed if there was a difference of at least 5mm between discs with and without clavulanic acid.

The absolute number of blood cultures collected fluctuates on an annual basis, but has approached 21000 per year since 2013. Of these, approximately 13,000 are from paediatrics and 8,000 from adults. The most commonly isolated pathogens are non-typhoidal Salmonellae, *Salmonella* Typhi and *Streptococcus pneumoniae* (estimated minimum incidence ≥300/year) followed by the other Enterobacterales, in particular *E.coli* and *Klebsiella* spp. (50–299/year) ^7^.

### Participant selection and enrolment procedures

Daily reviews of the blood culture bench in the MLW microbiology laboratory will be conducted on Monday to Friday, to identify consecutive blood cultures which are positive for pathogens of interest. Blood cultures which become positive over a weekend will be identified by the study team for recruitment on a Monday morning. Once the blood culture result is final, patients will be identified in the hospital and enrolled following informed consent, aiming for recruitment as soon as possible after the final blood culture result is known. If a patient has been discharged by the time the final blood culture result is available, they will be contacted for review and potential recruitment if contact details are available. If a patient has died by the time the final result is available, they will still be included in the study and their medical records will be collected for review and CRF completion.

At enrolment, a baseline questionnaire will be conducted, collecting demographic and clinical information including admission admission vital signs, pre-hospital healthcare attendance, any treatments including antibiotics administered, health-related quality of life (HRQoL) and a health-care utilisation survey. Pre-hospital healthcare information will be used to classify infections into community acquired or healthcare associated. Questionnaires will be completed by a combination of patient interview, medical note review, and guardian interview if the patient is a child or is obtunded. In patients who have died by the time the blood culture is identified, the same questionnaires will be completed via medical note review, with data recorded as missing if it is not available. Vital signs will be performed by the study nurse at enrolment and patients assessed by a study clinician who will review the admission history and carry out clinical history and examination where indicated.

All participants will be reviewed as soon as possible after recruitment, by the study PI (a specialist trainee in infectious diseases) or a clinical member of the study team and discussed with the PI. If required recommnedations on treatment and diagnostics will be made but as this is an observational study, the care of the patient will be directd by the clinical team responsible fot the patient.

### Sample collection

Blood samples will be collected from participants at enrolment and used to provide a set of baseline parameters that will aid in the clinical assessment of the participants illness. Blood will be tested for Full Blood Count (FBC) and creatinine and for CD4 count if HIV infected. Point of care tests will be carried out on capillary blood for capillary lactate (Lactate Pro 2, Arkray, Japan) and quantitative C-Reactive Protein (CRP) (CRP single test kit , used with the NycoCard II Reader, Abbott, UK, 1116078 and SBUK0028). HIV testing will be done as part of routine patient care, following Malawi national guidelines ^24^. If a patient’s HIV status is unknown at the time of recruitment, they will be referred for HIV testing and counselling via standard QECH pathways. Urine will be collected at enrolment for screening dipstick and cultured if the dipstick is positive for leucocytes or nitrites. One stool sample will be taken at enrolment, or a rectal swab if it is not possible for a patient to provide stool. All other sample collection including urinary lipoarabinomannan (uLAM), and Sputum Xpert are done at the discretion of the clinical team providing routine care for the patient.

### Follow up procedures

Patients will be followed up throughout their admission until discharge or death, allowing for measurement of in-hospital mortality. Information on treatments administered and vital signs will be recorded daily throughout the admission. To allow for survival analysis and calculation of 28-day and 6-month mortality, patients or their families will be telephoned at 28-days, three and six months post discharge. At follow-up, patients will be questioned to establish details of any antimicrobials received or healthcare facility usage since the last phone contact. If a patient dies, family members are asked the date of death.

### Classification of Gram-negative blood culture

A preliminary set of classifications will be developed to describe the impact of each positive blood culture on each participant, following the anecdotal observation that patients were surviving 3GC-R BSI episodes without receiving an antibiotic to which the organism is susceptible. These categories will initially be developed by the study PI (a specialist trainee in infectious diseases), and will be used to broadly classify each BSI episode into the following categories: definite Gram-negative sepsis, probable Gram-negative sepsis, transient or occult bacteraemia, or contaminated blood culture.

An expert panel, consisting of locally experienced physicians, adult and paediatric infectious disease specialists and a consultant microbiologist will then be assembled to pilot and finalise the classifications. This group will be presented with clinical vignettes for six patients and asked to anonymously classify each patient into the set of preliminary categories. An example of these vignettes, as they will be presented to the panel, are shown in Table 3. Any discrepancies in classifications will be discussed between the group and a final set of classifications and definitions decided. These classifications are shown in Table 4.

**Table 3.** Participant vignettes: three example participants discussed at the consensus meeting, shown with final decisions on patient classification.

**Participant-1** *E.coli* S: ceftriaxone, chloramphenicol, gentamicin, ciprofloxacin, meropenem R: ampicillin, cotrimoxazole **Classification:** **Definite Gram-negative sepsis**	26 year old female HIV negative, normally fit and well. 2 weeks postpartum. Caesarian section done at Queen Elizabeth Central Hospital. Unwell for 10 days post-operatively: abdominal pain, and fevers. Admission: Temperature 40.0°C. SIRS = Yes Recruitment Day 7: Temperature 37.0°C. SIRS= Yes Wound clean. No urinary catheters. Urine dipstick negative. Antibiotics: 9 days ceftriaxone, 5 days ciprofloxacin Bloods Day 7: WCC 8.3 x10 ^9^ L ^-1^, CRP 91 mg/L, Lactate 2.6, Outcome: Discharged alive
**Participant-2** *E.coli* S: ceftriaxone, chloramphenicol, gentamicin, ciprofloxacin, meropenem R: ampicillin, cotrimoxazole **Classification:** **Definite Gram-negative sepsis**	72 year old male HIV positive, on ART 10 years. Benign prostatic hyperplasia. Unwell for 3–4 weeks: confusion, cough, weight loss, lethargy. Pre-hospital: Co-amoxiclav and azithromycin within 1 month of admission. Week 2 TB Treatment Admission: Temperature 35.7°C, GCS 10. SIRS = Yes Recruitment Day 5: Temperature 37.5°C. SIRS = No Bloods Day 5: CD4 158 μL ^-1^, WCC 26.0 x10 ^9^ L ^-1^,CRP >120mg L ^-1^, lactate 3.4mmol L ^-1^, creatinine 862mmol L ^-1^ Antibiotics: Ceftriaxone 24 hours Outcome: Died in hospital
**Participant 3** *E.coli* S: chloramphenicol, gentamicin, amikacin, meropenem, co-amoxiclav R: ceftriaxone, ampicillin, cotrimoxazole, ciprofloxacin **Classification:** **Possible Gram-negative** **sepsis**	32 year old woman, HIV positive, ART 2 years. Mechanical fall into drain, pain in hip and hx of fevers 24 hours later. Associated headache and diarrhoea. Presented to Emergency Department after 5 days, had blood culture and outpatient follow-up arranged. No vital signs available on admission. Discharged on no antibiotics. Bloods Day 6: WCC 6.3 x10 ^9^ L ^-1^, creatinine 39mmol L ^-1^,CD4 847μL ^-1^, lactate 2.6mmol L ^-1^, CRP 36mg L ^-1^, urine dip negative Repeat blood culture on Day 6 = negative, no antibiotics in between Antibiotics: one dose of gentamicin on day 6, then discharged on nothing. Outcome: Discharged alive

**Abbreviations:** SIRS, systemic inflammatory response syndrome; ART, antiretroviral therapy; WCC, white cell count; CRP, C-reactive protein

**Table 4.** Classification scheme for Gram-negative blood cultures.

Category	Definition
**Definite Gram-negative** **sepsis consequent upon** **cultured isolate**	The blood culture isolate is contributing to the patient’s clinical state and treatment was considered to be required.
**Probable Gram-negative** **sepsis consequent upon** **cultured isolate**	The blood culture isolate is probably contributing to the patient’s clinical state, but there is insufficient evidence to confirm or refute this. Treatment was considered to be required
**Possible Gram-negative** **sepsis consequent upon** **cultured isolate**	The blood culture isolate may be contributing to the patient’s clinical condition, but the patient improved without antibiotics predicted to be active based on antimicrobial susceptibility testing, and it is not possible to confirm or refute definite/probably Gram-negative (GN) sepsis. i.e. Treatment was considered to be required but the patient improved without antibiotics likely to be active against the isolate.
**Occult or transient** **bacteraemia**	The blood culture isolate may have contributed to the patient’s clinical condition, but by the time they are assessed with the culture, they have improved. Unlike the definition for ‘possible GN sepsis’, treatment was not considered to be required, but instead a repeat blood culture was desirable.
**Definite contaminant**	The isolate has never contributed to patient’s condition and was very likely not present in the bloodstream
**Probable contaminant**	The isolate probably never contributed to patient’s condition and was probably not present in the bloodstream/or there is insufficient evidence to say for sure.

On completion of recruitment, the first 50 study participants will be presented to the expert panel and classified into definite/probable or possible Gram-negative sepsis, transient or occult bacteraemia, or definite/probably contaminant ( Table 4). For neonatal patients, a consultant neonatologist will be included. The panel will first be asked to anonymously categorise the participants and individual responses will be recorded. Any discrepancies will be discussed and resolved by consensus. Following review and classification of the first 50 participants, the panel will re-assess the classification process to decide if sufficient clinical and laboratory data are available to confidently classify patients in this manner. At a minimum, patients will be objectively defined on the basis of:

1. Having a severe inflammatory response syndrome (SIRS) or not;
2. Their treatment response (clinical improvement based on factors such as resolution of symptoms and signs including fever, with or without active antibiotics or died with or without active antibiotics).

The lack of robust and universally validated sepsis severity score applicable to a cohort of both adults and children in a low-income setting ^25^, means that SIRS criteris will be used as a minimum to help classify patients, alongside other clinical and biochemical parameters.

In addition, the panel will be asked for consensus on likely focus of clinical infection in patients who are considered to have a definite/probable or possible Gram-negative sepsis ( Table 5). The panel will be asked to classify into likely rather than definite focus, because of a desire not to overclassify non-focal sepsis in a setting were lack of diagnostic resources frequently limit the ability to definitively confirm focus of infection.

**Table 5.** Clinically suspected focus of infection.

Focus
Non-focal Central-line associated Clear focus of infection • Urinary tract infection • CNS • Skin and soft tissue • Gastrointestinal (hepatobiliary) • Gastrointestinal (non hepatobiliary) • Cardiovascular system • Respiratory tract infection (other than VAP) • Reproductive tract infection • Surgical site infection • Bone and joint • Post-operative, non-focal • VAP Unknown Other

**Abbreviations:** CNS, central nervous system; VAP, ventilator associated pneumonia

### Data analysis plan for primary outcomes

The primary outcomes for the study are mortality and morbidity from 3GCR-E. The effect of 3GCR-E on mortality and discharge alive will be estimated using a logistic regression model for 28-day mortality and a Cox proportional hazards model for time to death, adjusting for confounders such as patient co-morbidities. In the first instance, these models will be fitted to mortality data from the complete cohort. Uncertainty in true bloodstream infection status will then be explored using a Bayesian latent class model which incorporates the output of the classifications as a latent variable. Taken together with BSI incidence estimates from Blantyre ^7^, this will allow estimation of mortality from BSI in the Blantyre, assuming that cases admitted to QECH are representative of the general population. We will use hospital length of stay as a proxy outcome measure for morbidity ^8^. To estimate length of hospital stay associated with 3GC-R-E, we will use multistate modelling, with time from hospital admission as the time scale ^26^. Statistical analyses will be conducted using R (R Foundation for Statistical Computing, Vienna, Austria).

### Health economic components

The health economic data collection will allow for three types of comparisons between patients with 3GC-R and 3GC-S BSI: healthcare provider costs, costs incurred by patients and their families as a result of hospitalisation, and health-related quality of life (HRQoL). Primary costing studies and data capture tools described below have been developed and validated for adult inpatients only ^27^, therefore children under 18 are excluded from this component of the study.

***Healthcare provider costs***. Upon discharge or death, information from the patient’s medical record will be extracted by a study clinician, to establish the medications and dosages given, duration of hospital admission, types and numbers of investigations and procedures performed and the participant’s outcome. Costs of these healthcare resources will be derived from a previous primary costing study undertaken at QECH ^27^. The international market price will be used to estimate costs for all medication given ^28^.

***Direct non-medical and indirect costs***. Questionnaires will be administered to patients and their guardians as soon as possible after recruitment. All questionnaires are provided as extended data ^29^. Data collected will include cost of transportation, food, drinks, toiletries, clothing and other items bought during the hospital admission. For indirect costs, any time off work taken by participants or their guardians is recorded together with self-reported income. The development and language translations of these questionnaires followed previous procedures ^27, 30^.

***Health-related quality of life***. The Chichewa version of the EuroQoL EQ-5D-3L will be used to assess HRQoL of participants at recruitment and discharge from hospital as well as at 28-day follow-up ^31^. The EQ-5D has a descriptive component asking participants to rate their health status across a number of domains and a visual analogue scale (VAS) similar to a thermometer, and ranges from 100 (best imaginable health state) to 0 (worst imaginable health state). EQ-5D utility scores will be derived from responses to the descriptive components using the Zimbabwean EQ-5D tariff set ^32^.

Mean differences in total direct health provider cost, total direct non-medical and indirect cost and HRQoL outcomes between participants with 3GC-R and 3GC-S BSI will be estimated. Non-parametric bootstrap methods will be used to account for possible skewness in distribution of economic data. Multivariable analysis will be undertaken to explore the independent effects 3GC-R on these economic costs and HRQoL outcomes. For HRQoL outcomes, EQ-5D utility and VAS scores will also be compared between participants at recruitment, discharge and follow-up where data are available.

### Data capture and storage

Data will be collected using Open Data Kit software (ODK, 1.4.10) and TeleForm Data Capture Software (10.7). Completed ODK forms are pushed daily to a dedicated secure SQL database. Teleform paper forms will be checked, scanned and validated by the MLW data team, in discussion with the clinical team if required and validated TeleForm data pushed to the SQL database. Completed paper TeleForm records will be stored securely in the MLW data department. All data on the study database will be stored securely with access restricted to the study PI and the database administrators in the MLW data department. Results of laboratory investigations in the MLW laboratory will be stored in the MLW PreLink laboratory information management system (LIMS), anonymised and linked only to the participant unique study ID number.

### Sample size considerations

The study is powered to detect a difference in 28-day mortality rates between participants with 3GC-R and 3GC-S BSI. There are no studies from sSA powered to detect mortality from ESBL BSI, on which to guide our sample size estimates, but a large multi-centre European study found that mortality was 14% higher in patients who had an ESBL positive BSI versus those who had ESBL negative BSI ^33^. Based on this, we aim to recruit 250 patients to the cohort, which would provide 80% power to detect a difference in 28-day mortality rates of 10% vs. 24.1%. If this recruitment target is not achieved, a more modest 200 patients would still provide 80% power to detect mortality of 10% vs 25.8%. These calculations assume a 50:50 split in 3GC-R and 3GC-S infections, based on 2016 figures ^7^. An imbalance in this split will have minimal impact on the statistical power of the study. We are aiming to recruit 250 patients who have definite, probable or possible Gram-negative sepsis, therefore have inflated the overall sample size to 350 participants given that some patients will be censored from the study following expert case review.

### Ethics

Ethical approval for the study was granted by the Malawi College of Medicine Research Ethics Committee (COMREC), protocol number P.10/17/2299 and by the Liverpool School of Tropical Medicine Research Ethics committee, protocol number 17-063. LSTM acted as the study sponsor. Written informed consent is obtained from study participants, the participant’s parent/guardian if they are a child aged 18 years, or from a guardian if the patient lacks capacity to consent. Written informed consent is obtained from study participants, the participant’s parent/guardian if they are a child aged 18 years, or from a guardian if the patient lacks capacity to consent. See below for explanation of consent for patients who have died. In addition to parental consent, assent was sought from children aged eight years and above, in accordance with WHO guidelines and the requirements of the local ethics committee ^34^.

As consent was not possible for patients who had died by the time of enrolment, all medical records were anonymised by the study clinical officer, who was a Malawi Ministry of Health employee, at the request of COMREC. Once recruited, no other member of the study team had access to any identifiable medical records for these patients.

### Dissemination

Results from this study will be presented internally within the College of Medicine and QECH, Malawi College of Medicine Research Ethics Committee and disseminated to the Ministry of Health, Malawi. Manuscripts will subsequently be prepared for publication in peer-reviewed journals, which will be made freely available via open-access publication.

### Study status

Recruitment to the study is currently ongoing and is expected to be completed in March 2020.

## Discussion

This study is designed to investigate the attributable morbidity, mortality and economic cost of third-generation cephalosporin resistant bloodstream infections in Malawi, a country which has the largest bacteraemia and AMR surveillance dataset from sSA, but in which the health burden of AMR infections is currently unknown. We aim to address this knowledge gap by assessing the healthcare burden of resistance to one of the most commonly used and frequently last-line antibiotics in hospitalised inpatients in Malawi.

Our approach of prospective recruitment and detailed characterisation of all BSI episodes will generate reliable data on the impact of bloodstream infection on patients, and in turn, the burden of 3GC-R infection. In line with recently published guidance on quality reporting of AMR data ^35^, we will be able to provide a clear account of microbiological sampling criterial, sampling frame and laboratory methods, as well as clinical metadata including empiric antibiotic regimens, HIV status and healthcare attendance.

This study is limited to one hospital but it is hoped that these data will be used to generate accurate burden estimates for Malawi, and that the methods will be replicated by future investigators wishing to generate robust data on the impact of drug-resistant infections. By estimating attributable mortality, morbidity and economic cost in a prospective cohort, we will generate high quality data that will be amongst the first of their kind from sSA and that will consequently be able to inform global burden of disease estimates

## Data availability

### Underlying data

No data are associated with this article

### Extended data

Zenodo: Case report forms (CRFs) used for the publication: Estimating the burden of antimicrobial resistance in Malawi: protocol for a prospective observational study of the morbidity, mortality and economic cost of third-generation cephalosporin resistant bloodstream infection. http://doi.org/10.5281/zenodo.3634466 ^29^

This project contains the following extended data:

- CRFs_Wellcome.pdf (Study questionnaires)

Data are available under the terms of the Creative Commons Attribution 4.0 International license (CC-BY 4.0).
