# Supplementary material for: Estimating the burden of antimicrobial resistance in Malawi: protocol for a prospective observational study of the morbidity, mortality and economic cost of third-generation cephalosporin resistant bloodstream infection
Source: Wellcome Open Res. 2020 Jun 1;5:29. Originally published 2020 Feb 13. [Version 2] doi: 10.12688/wellcomeopenres.15719.2 (PMC7290274; doi:10.12688/wellcomeopenres.15719.2)
Supplement: Peer review history

# Peer review: Reviewer response for version 2

**DOI:** 10.21956/wellcomeopenres.17555.r38912

**Published:** 2020-06-11

**Authors:** Priscilla Rupali

**Affiliations:** 1 Infectious Diseases Training and Research Center, Christian Medical College, Vellore, India

I am happy with the revisions and explanations provided. I give my approval for indexing.

Is the study design appropriate for the research question?

Yes

Is the rationale for, and objectives of, the study clearly described?

Yes

Are sufficient details of the methods provided to allow replication by others?

Partly

Are the datasets clearly presented in a useable and accessible format?

Partly

Reviewer Expertise:

Infectious Diseases.

I confirm that I have read this submission and believe that I have an appropriate level of expertise to confirm that it is of an acceptable scientific standard.

# Peer review: Reviewer response for version 2

**DOI:** 10.21956/wellcomeopenres.17555.r38913

**Published:** 2020-06-09

**Authors:** Jan Jacobs, Bieke Tack

**Affiliations:** 1 Institute of Tropical Medicine, Antwerp, Antwerp, Belgium; 2 Department of Clinical Sciences, Unit of Tropical Laboratory Medicine, Institute of Tropical Medicine Antwerp, Antwerp, Belgium

A non-blocking comment from our side is about the qSOFA versus the SIRS score: as the authors have written in their reply to our comments, a sepsis classification system is extremely challenging in a low-resource setting. However, we do not agree that the cited letter to the editor ^1^ provides enough evidence to state that qSOFA score is not likely to be useful for clinical management or research in a low-resource setting. The letter questions the superiority of the qSOFA score to the SIRS score, which was assessed by Rudd *et al.* in ten low- and middle income countries ^2^. They state that the data from Rudd *et al*. are too biased for comparison of the predictive performance between the qSOFA and SIRS score. However, even so, Rudd *et al*. provide interesting data that support the use of qSOFA as a bedside clinical decision-making tool. In contrast to the SIRS score, the qSOFA score does not need laboratory testing and is a less technically challenging alternative for the low-resource context.

Is the study design appropriate for the research question?

Partly

Is the rationale for, and objectives of, the study clearly described?

Yes

Are sufficient details of the methods provided to allow replication by others?

Partly

Are the datasets clearly presented in a useable and accessible format?

Not applicable

Reviewer Expertise:

Clinical Bacteriology.

We confirm that we have read this submission and believe that we have an appropriate level of expertise to confirm that it is of an acceptable scientific standard.

## References

[1] : Validity of the qSOFA Score in Low- and Middle-Income Countries. *JAMA*.2018;320(19) : 10.1001/jama.2018.14473 10.1001/jama.2018.14473 30458485

[2] : Association of the Quick Sequential (Sepsis-Related) Organ Failure Assessment (qSOFA) Score With Excess Hospital Mortality in Adults With Suspected Infection in Low- and Middle-Income Countries. *JAMA*.2018;319(21) : 10.1001/jama.2018.6229 10.1001/jama.2018.6229 PMC613443629800114

# Peer review: Reviewer response for version 1

**DOI:** 10.21956/wellcomeopenres.17231.r38060

**Published:** 2020-04-27

**Authors:** Priscilla Rupali

**Affiliations:** 1 Infectious Diseases Training and Research Center, Christian Medical College, Vellore, India

The idea is definitely good and will provide meaningful data which will likely help in investment of resources in bringing AMR down. 

**Overview**: This study protocol attempts to in a prospective manner compare mortality, morbidity and economic impact between infections which are caused by 3 ^rd^ GC resistant vs sensitive GNB *E.coli* and *Klebsiella spp*. Data regarding this is available from high income countries and they hope to derive this information in sub Saharan Africa.

 Ethical approval has been granted for the study.

 Comments:

**Study Approach**: In this section, the authors have described the general approach and various strength of their study. I agree with the authors that a prospective study studying this is important and may provide new contextual information relevant to the specific clinical setting in SSA.

- Classifying patients by experts may not be required if the definitions set out for definite, probable and transient BSI are followed by the study team for recruitment. I am not sure, review by experts adds anything to this.
- The primary outcomes are not well defined and I presume are mortality, LOS as predictor of morbidity and costs.
- Counterfactual approach – not validated terminology and is best excluded as it confuses the readers.
- Severity of disease needs to be better defined by a validated score qSOFA or APACHE rather than SIRS as it is likely to have bearing on the primary outcome.
- "If Enterobacterales are genuinely present in blood cultures as contaminants or cryptic organisms in significant numbers, it would have profound implications for burden of AMR studies." - I am not sure I agree with this. If a positive blood culture is classified as a contaminant – in the absence of corroborating clinical features, antibiotics are unlikely to be given and this contribution to AMR burden is unlikely to be significant.

**Data Analysis**: “Uncertainty in true bacterial infection will be explored by a latent variable approach and a bayesian paradigm”. Generally this approach is used for a diagnostic test where there is no established gold standard. It is not applicable here.

- It is unclear exactly what variables are being collected which could impact the primary outcome of death in the 2 groups.
- Antibiotics - appropriateness, duration, empirical antibiotics given or not are not mentioned.
- Primary outcomes and variables affecting that are not mentioned in coherent detail.
- Appreciate that economic and HRQoL outcomes have been mentioned in a fair bit of detail.
- It has been mentioned "In patients who have died by the time the blood culture is identified, the same questionnaires will be completed via medical note review, with data recorded as missing if it is not available" - will these missing data be included in the analysis?

**Participant Selection and Enrollment Procedures**:

- Table 1 - " **Patient **speaks neither English or Chichewa" - is an exclusion criteria but it is unclear what happens when recruiting children or if patient passes away before blood culture is obtained.
- "If a patient has died by the time the final result is available, their medical records will be collected for review" - it is unclear how informed consent will be obtained from a family member .

**Follow-up Procedures**: "If a patient dies, family members are asked the date of death", thus this mean only date of death or details as mentioned earlier regarding antimicrobial therapy or healthcare facility admissions? Kindly clarify.

Is the study design appropriate for the research question?

Yes

Is the rationale for, and objectives of, the study clearly described?

Yes

Are sufficient details of the methods provided to allow replication by others?

Partly

Are the datasets clearly presented in a useable and accessible format?

Partly

Reviewer Expertise:

Infectious Diseases.

I confirm that I have read this submission and believe that I have an appropriate level of expertise to confirm that it is of an acceptable scientific standard, however I have significant reservations, as outlined above.

# Response

**Published:** 2020-05-23

**Authors:** Rebecca Lester

**Affiliations:** Liverpool School of Tropical Medicine, UK

**Comments:**

**Study Approach: In this section, the authors have described the general approach and various strength of their study. I agree with the authors that a prospective study studying this is important and may provide new contextual information relevant to the specific clinical setting in SSA.**

· **Classifying patients by experts may not be required if the definitions set out for definite, probable and transient BSI are followed by the study team for recruitment. I am not sure, review by experts adds anything to this.**

Many thanks for this feedback. We believe that the expert panel review is essential to the classifications process as expertise in local infectious disease management is important when making decisions surrounding the complex patients in this setting. Infectious disease clinicians are experienced in making decisions about the need for antibiotics in patients with a positive blood culture whereas the study team are not. Additionally, at the point of recruitment it is often the case that not all the required clinical information (such as blood tests) are available, and these need to be collated for these discussions. 

The study team will reflect on whether this was a useful exercise post-hoc in the results manuscript.

**The primary outcomes are not well defined and I presume are mortality, LOS as predictor of morbidity and costs.**

We have added a sentence to the data analysis section as follows:

*The primary outcomes for the study are mortality and morbidity from 3GCR-E.*

**

**Counterfactual approach – not validated terminology and is best excluded as it confuses the readers.**

Please also see our response to reviewer 1’s comments on the counterfactual approach. We have chosen to keep this terminology, which is a standard approach for causal inference in epidemiological studies, but expanded on our explanation of the term and included further references, including to the article written by members of the Surveillance and Epidemiology of Drug-resistant Infections Consortium (SEDRIC) consortium, which describes the importance of the counterfactual approach in AMR research. 

**Severity of disease needs to be better defined by a validated score qSOFA or APACHE rather than SIRS as it is likely to have bearing on the primary outcome.**

Please also see our response to reviewer 1’s comments, with regards to the difficulties with these scores. Notably, qSOFA is not validated in low income settings[1], nor in paediatric populations, and APACHE scores require parameters which are not available in our study setting, such as serum sodium and potassium. SIRS has been chosen because the required parameters are readily available, but will simply be used as a minimum criteria for classifications, which will also take into account other variables such as CRP and lactate. 

**"If Enterobacterales are genuinely present in blood cultures as contaminants or cryptic organisms in significant numbers, it would have profound implications for burden of AMR studies." - I am not sure I agree with this. If a positive blood culture is classified as a contaminant – in the absence of corroborating clinical features, antibiotics are unlikely to be given and this contribution to AMR burden is unlikely to be significant.**

Many thanks for this feedback and we agree that this is a complex area. However, in the majority of cases, and particularly in Malawi, Enterobacterales are rarely if ever considered contaminants by clinicians and are invariably treated with active antibiotics where they are available. Further, data from diagnostic microbiology laboratories are frequently reported to ministries of health and thence to GLASS as aggregate data, without any reference to clinical metadata beyond that which is on the request form. The lack of standardisation of how microbiological data are captured is a major problem in assessing the global burden of drug resistant infection, which may well lead to overestimates. It is the prospective nature of this study and expert review of all cases that allows the distinctions between contaminants and true infections to be made and we believe this is a strength of the study.  

**Data Analysis** **: “Uncertainty in true bacterial infection will be explored by a latent variable approach and a bayesian paradigm”. Generally this approach is used for a diagnostic test where there is no established gold standard. It is not applicable here.**

Please also see our response to Reviewer 1’s comments. A Bayesian latent class model will be used to explore uncertainty in true BSI status given the clinical classifications decided by the expert panel.  This is a standard approach for dealing with uncertainty in models from clinical observational studies in addition to studies evaluating diagnostic tests[3].

**It is unclear exactly what variables are being collected which could impact the primary outcome of death in the 2 groups.**

Unfortunately, due to space limitations we are unable to describe all of these variables fully in the text. However, the study CRFs are included as extended data and referenced in the main paper. For example, we will collect data on patient co-morbidities, baseline vital signs and previous healthcare exposures – all of which could be acting as confounders in outcome models. 

**Antibiotics - appropriateness, duration, empirical antibiotics given or not are not mentioned.**

As this is an observational study, these will depend on the clinical care provided by the patient’s team and will not be prescribed as part of the study. We have clarified this in the text (Methods/Participant selection and enrolment procedures) (please also see response to reviewer 1’s comments). 

**Primary outcomes and variables affecting that are not mentioned in coherent detail**

We have now clarified the primary outcomes. As mentioned above, study CRFs with all variables collected are included as extended data.

**Appreciate that economic and HRQoL outcomes have been mentioned in a fair bit of detail.**

We thank the reviewer for their support

**It has been mentioned "In patients who have died by the time the blood culture is identified, the same questionnaires will be completed via medical note review, with data recorded as missing if it is not available" - will these missing data be included in the analysis?**

Any data that are available from patients who have died will be included, and missing data will be accounted for in the final analysis through a process of multiple imputations. 

**Participant Selection and Enrollment Procedures**:

**Table 1 - "Patient speaks neither English or Chichewa" - is an exclusion criteria but it is unclear what happens when recruiting children or if patient passes away before blood culture is obtained.**

Please see the Ethics section  for an explanation of consent in children. We have added a sentence about assent, so the paragraph now reads:

*Written informed consent is obtained from study participants, the participant’s parent/guardian if they are a child aged 18 years, or from a guardian if the patient lacks capacity to consent. See below for explanation of consent for patients who have died.* *In addition to parental consent, assent was sought from children aged eight years and above, in accordance with WHO guidelines and the requirements of the local ethics committee.*

**"If a patient has died by the time the final result is available, their medical records will be collected for review" - it is unclear how informed consent will be obtained from a family member .**

An ethical waiver for informed consent was obtained from the local Research Ethics Committee. Instead, files of patients who have files are anonymised by a hospital employee and then reviewed by a member of the study team. We have added the following paragraph:

*As consent was not possible for patients who had died by the time of enrolment, all medical records were anonymised by the study clinical officer, who was a  Malawi Ministry of Health employee, at the request of COMREC. Once recruited, no other member of the study team had access to any identifiable medical records for these patients.*

**Follow-up Procedures** **: "If a patient dies, family members are asked the date of death", thus this mean only date of death or details as mentioned earlier regarding antimicrobial therapy or healthcare facility admissions? Kindly clarify.**

Family members are asked date of death in addition to the other questions asked to surviving patients at follow-up. 

1  Lewis JM, Henrion M, Rylance J. Validity of the qSOFA Score in Low- and Middle-Income Countries. JAMA **2018**; 320(19): 2038-9.

2  Barrasa-Villar JI, Aibar-Remon C, Prieto-Andres P, Mareca-Donate R, Moliner-Lahoz J. Impact on Morbidity, Mortality, and Length of Stay of Hospital-Acquired Infections by Resistant Microorganisms. Clin Infect Dis **2017**; 65(4): 644-52.

3  Deloria Knoll M, Fu W, Shi Q, et al. Bayesian Estimation of Pneumonia Etiology: Epidemiologic Considerations and Applications to the Pneumonia Etiology Research for Child Health Study. Clin Infect Dis **2017**; 64(suppl_3): S213-S27.

# Peer review: Reviewer response for version 1

**DOI:** 10.21956/wellcomeopenres.17231.r37923

**Published:** 2020-03-02

**Authors:** Jan Jacobs, Bieke Tack

**Affiliations:** 1 Institute of Tropical Medicine, Antwerp, Antwerp, Belgium; 2 Department of Clinical Sciences, Unit of Tropical Laboratory Medicine, Institute of Tropical Medicine Antwerp, Antwerp, Belgium

The paper describes a study protocol used to assess the impact (mortality, direct and indirect costs and other healthcare outcomes) compared between patients infected with ceftriaxone-resistant versus ceftriaxone-susceptible *Enterobacteriales* bloodstream infections. The study protocol has already been granted ethical approval.

 Major comments

- The authors correctly observed that patients with clinical suspicion of sepsis and culture-proven bloodstream infection caused by third generation cephalosporin resistant *Enterobacteriales *survived despite treatment with non-effective antibiotics. In addition to " *question the role of the bacterial isolate playing in the patient's presentation*", there is the fact that also in the pre-antibiotic era, a proportion of patients survived bloodstream infections – see Friedman (2016) ^1^. So the reasoning of "survived = probably contaminant" may not be apply in all situations, *e.g.* not in settings with less severe symptoms and correctable causes such as urinary catheter-related bacteremia. It would be good to address this in the Discussion session. 
- P3: it is not clear how data of patients who died are processed *"…by reviewing every patient alive by the time their culture is positive*" means that patients who died with 24h – 48h (before expected growth of blood cultures) are not included? This may impact the study results, as – depending on the referral track and type of patients (children) – mortality may be highest in the first 24h upon admission. However, on P4 it is stated that " *If a patient* *has died by the time the final result is available, their medical records will be collected for review*".
-  P5: the authors may have good reasons to stick to "severe SIRS" but it I suggest to address these reasons (e.g. why not using the qSOFA) or any other gradable disease severity classification applicable to adults, children and neonates– this may be challenging. Is there any use of the lactate or CRP results (one or serial measurements)?
- P5 statistics: I am not an expert in this domain. My main comment is: " *latent variable approach and a Bayesian paradigm*" is a bit vague, do the authors mean they will use both approaches in 1 model (e.g. Bayesian Latent Variable Modeling) or build 2/more models (Bayesian stats, structural equation modelling,...) and compare them? Further, it is not clear how they will build the latent variable, will it be built on the criteria that will used for the classification? Is there any validation to assume the length of hospital stay as a proxy for morbidity? It would be good to assess also how differences in co-morbidity (and their effect on the outcomes) are taken into account. 
- P5: suggest to specify how " *got better*" - Fever resolution, discharge, symptom resolution…
- P4 Representativeness: blood culture sampling indications may not be strictly followed, with possibilities to under- and over-sampling (see P4) – is a "screening log" applied to allow estimation of the non- or incorrectly sampled patients?
- P5 Clinical follow-up: both in the paragraph on recruitment and follow-up procedures, I miss data about treatment guidelines and collection of treatment data (including correct dosage in children). If I understand it correctly, not only the patients treated with ceftriaxone will be enrolled? Is there any classification according to (available and affordable) treatment alternatives in case of ceftriaxone-resistance? Is there a hospital policy about choice of second- and third-line antibiotics? 
- P7 Table 3: I suggest to take into account the issue of healthcare-associated infections, for which certain *Enterobacteriales* ( *Klebsiella, Enterobacter, Serratia, Citrobacter…)* and Acinetobacter may be more likely to involved (transient bacteremia; pseudo-bacteremia) than for instance *E. coli* which is more likely to be endogenous. 
- P7 Table 4: some of the " *clear focus*" may be difficult to assess – for instance "Urinary Tract Infection" and the "Respiratory tract infection" and "VVAP" 
- P6 – P7: Clinical care and follow-up: it is not clear at which moment the patients will be assessed and by who: if the assessment is done by a trained panel or experts, one can expect sensible and relevant therapeutic and diagnostic actions during the hospital course (e.g. extra cultures at surgical debridement, additional x-ray...). They may influence patient outcome. How will this be handled? 
- Table 1: Not clear why Acinetobacter is included – in terms of habitat and behavior, it is different from *Enterobacteriales* as well as from the other Gram-negative fermentative rods.
- P3: the "counterfactual" approach is not well described for naïve readers – this should be addressed as it is key to the approach.
- *"Blood cultures which are positive over a weekend will be identified on Monday morning" *meaning that a 2-day delay in identification and antibiotic susceptibility testing will occur and that directed treatment will be delayed – this can be important in situations where empiric treatment did not include effective treatment against Enterobacteriales – is this an issue or is ceftriaxone a part of the standard empiric treatment?

Minor comments

- Sensitive: suggest to replace by "Susceptible".
- Study Site: for reasons of representativeness, comparison and generalizability, some national demographic and health data could be provided (aside from the GDP).
- Study Site: why are only the admission data for the MJC supplied (and not those for the entire hospital?
- Study Site: I suggest to give an overview of the (estimated) distribution adult  versus children blood culture samples as well as community versus hospital-associated infections.
- Diagnostic bacteriology: blood volumes sampled are (too) low: this is a reality in low-resource settings but its implications should be discussed – it is possible that mainly patients with high bacterial loads will be selected and included. 
- Diagnostic bacteriology: not clear which antibiotics will be tested and of ESBL testing is done.
- Clinical presentation P5: not clear what is meant with " *admission physiology*": vital signs, clinical signs and symptoms, illness severity,...?
- The Abstract might be restructured to give more essential and detailed study information such as the (i) concrete classification of the role of the isolated bacteria and (ii) the healthcare economic data.
- Abstract: Some abbreviations not explained previously (3GC-S) and there is some inconsistency (3GCR-E versus 3GC-R) - probably it will be easier if no abbreviations are used.
- In line with the Keywords, "Africa" (L3) might be changed to "sub-Saharan Africa".

- References: Some references are (too) briefly cited (e.g. ref. 15 "World Bank). Suggest to add websites + date of access where appropriate.

- Consistency of abbreviations or unusual abbreviation: 3GCR-E versus 3GC-R and DRI – only used a few time in the text.
- Style: P4 and elsewhere: paragraph of methods mixes past, future and present tense.

Is the study design appropriate for the research question?

Partly

Is the rationale for, and objectives of, the study clearly described?

Yes

Are sufficient details of the methods provided to allow replication by others?

Partly

Are the datasets clearly presented in a useable and accessible format?

Not applicable

Reviewer Expertise:

Clinical Bacteriology

We confirm that we have read this submission and believe that we have an appropriate level of expertise to confirm that it is of an acceptable scientific standard, however we have significant reservations, as outlined above.

## References

[1] : The negative impact of antibiotic resistance. *Clin Microbiol Infect*.2016;22(5) : 10.1016/j.cmi.2015.12.002 416-22 10.1016/j.cmi.2015.12.002 26706614

# Response

**Published:** 2020-05-23

**Authors:** Rebecca Lester

**Affiliations:** Liverpool School of Tropical Medicine, UK

**The paper describes a study protocol used to assess the impact (mortality, direct and indirect costs and other healthcare outcomes) compared between patients infected with ceftriaxone-resistant versus ceftriaxone-susceptible *Enterobacteriales*bloodstream infections. The study protocol has already been granted ethical approval.**

****

**Major comments**

**The authors correctly observed that patients with clinical suspicion of sepsis and culture-proven bloodstream infection caused by third generation cephalosporin resistant *Enterobacteriales *survived despite treatment with non-effective antibiotics. In addition to " *question the role of the bacterial isolate playing in the patient's presentation*", there is the fact that also in the pre-antibiotic era, a proportion of patients survived bloodstream infections – see Friedman (2016)** **^1^** **. So the reasoning of "survived = probably contaminant" may not be apply in all situations, *e.g.* not in settings with less severe symptoms and correctable causes such as urinary catheter-related bacteremia. It would be good to address this in the Discussion session.**

Many thanks for this feedback and reference. Patients will not automatically be classified as probable contaminant if they survive the episode, but classified based on discussion of the case by the panel, taking into account all available information on each patient – including but not limited to their treatment response. For example, if a patient recovers because of source control (e.g. catheter removal) this would be noted by the panel and taken into account. The definition of “probable contaminant” in Table 3, is “ *the isolate probably never contributed to patient’s condition and was probably not present in the bloodstream/or there is insufficient evidence to say for sure.”*This definition allows for a more nuanced approach which accounts for all available parameters and the clinical judgement from the panel. The criteria are described in Table 4.

**P3: it is not clear how data of patients who died are processed *"…by reviewing every patient alive by the time their culture is positive*" means that patients who died with 24h – 48h (before expected growth of blood cultures) are not included? This may impact the study results, as – depending on the referral track and type of patients (children) – mortality may be highest in the first 24h upon admission. However, on P4 it is stated that " *If a patient has died by the time the final result is available, their medical records will be collected for review*".**

All patients who have died before the final blood culture result is available are screened and included in the study if possible. Information from their medical notes is recorded in the study CRFs via a retrospective note review. We have clarified this by removing the sentence “ *…by reviewing every patient alive by the time their culture is positive”*and will expand the sentence on page 4 to read:

*If a patient has died by the time the final result is available, they will still be included in the study and their medical records will be collected for review and CRF completion.*

**

**P5: the authors may have good reasons to stick to "severe SIRS" but it I suggest to address these reasons (e.g. why not using the qSOFA) or any other gradable disease severity classification applicable to adults, children and neonates– this may be challenging. Is there any use of the lactate or CRP results (one or serial measurements)?**

We agree that a sepsis classification system applicable to this cohort is extremely challenging and a universal grading system which is validated across all age groups in a low-income setting, is not available. qSOFA scores, for example, are not validated in LMICs and are not likely to be useful for clinical management or research in this setting[1]. We have chosen SIRS therefore, as a *minimum *criteria for objective classification. However, when the panel reviews the clinical vignettes, all available information, including both lactate and CRP, will be taken into account when deciding if a patient has sepsis. We now explain this approach further on P5, with the following text:

*The lack of robust and universally validated  sepsis severity score applicable to a cohort of both adults and children in a low-income setting* *[1]* *, means that SIRS criteria will be used as a minimum to help classify patients, alongside other clinical and biochemical parameters.*

**P5 statistics: I am not an expert in this domain. My main comment is: " *latent variable approach and a Bayesian paradigm*" is a bit vague, do the authors mean they will use both approaches in 1 model (e.g. Bayesian Latent Variable Modeling) or build 2/more models (Bayesian stats, structural equation modelling,...) and compare them? Further, it is not clear how they will build the latent variable, will it be built on the criteria that will used for the classification? Is there any validation to assume the length of hospital stay as a proxy for morbidity? It would be good to assess also how differences in co-morbidity (and their effect on the outcomes) are taken into account.**

This will be one model –which takes a Bayesian latent class approach. This approach is simply a method to explore the uncertainty in each patient’s true BSI status, acknowledging that it is error-prone. The latent variable will incorporate the output of the classifications. We have altered the text to make this clearer:

*Uncertainty in true bloodstream infection status will then be explored using a Bayesian latent class model which incorporates the output of the classifications as a latent variable.*

Any confounders such as co-morbidities will be conditioned upon in the mortality models. We have clarified this in the text with an additional sentence in the data analysis section. 

Hospital length of stay is a frequently used as a proxy outcome measure of patient morbidity. Other morbidity outcome measures such as ICU admissions, need for surgery, re-hospitalisation and disability status at discharge or follow-up, are more variably used, and not always applicable to low-income settings[2]. We have included an additional reference for this and clarified this in the text.  

**P5: suggest to specify how " *got better*" - Fever resolution, discharge, symptom resolution…**

We have changed this to “clinical improvement based on factors such as resolution of symptoms and signs including fever”. We have not defined specific criteria as these may not be applicable to the entire cohort. 

**P4 Representativeness: blood culture sampling indications may not be strictly followed, with possibilities to under- and over-sampling (see P4) – is a "screening log" applied to allow estimation of the non- or incorrectly sampled patients?**

We are reliant on blood cultures obtained through the routine clinical service at QECH. Given the huge numbers of patients presenting to the hospital daily, it is unfortunately not logistically possible to have a screening log for all potentially eligible patients presenting to the Emergency Department, nor would a screening log adequately capture the reasons a clinician chose to culture, or not to culture any given patient. This is a potential limitation of the data which we will have the opportunity to discuss following analysis. 

**P5 Clinical follow-up: both in the paragraph on recruitment and follow-up procedures, I miss data about treatment guidelines and collection of treatment data (including correct dosage in children). If I understand it correctly, not only the patients treated with ceftriaxone will be enrolled? Is there any classification according to (available and affordable) treatment alternatives in case of ceftriaxone-resistance? Is there a hospital policy about choice of second- and third-line antibiotics?**

It is correct that all patients will be enrolled, regardless of treatment as this is an observational study with no treatment interventions. We have added sentences to the recruitment and follow-up paragraphs, to clarify that information on treatments administered will be collected throughout the patient’s hospital stay and this information may be incorporated into outcome models if appropriate. The lack of available alternatives to ceftriaxone means that policies on second- and third-line antibiotics cannot currently be incorporated into antibiotic guidelines. We have added a sentence to the introduction to explain this.

**P7 Table 3: I suggest to take into account the issue of healthcare-associated infections, for which certain *Enterobacteriales* ( *Klebsiella, Enterobacter, Serratia, Citrobacter…)*and Acinetobacter may be more likely to involved (transient bacteremia; pseudo-bacteremia) than for instance *E. coli* which is more likely to be endogenous.**

Thank you for this suggestion, we agree this is an important component of the study. Information on epidemiological attribution of infections (community vs healthcare-associated) will be collected as part of the study so we will be able to take these factors into account during the classifications. We have clarified that we will collect this information with the following sentence in Methods.

*Pre-hospital healthcare information will be used to classify infections into community acquired or healthcare associated.*

**

**P7 Table 4: some of the " *clear focus*" may be difficult to assess – for instance "Urinary Tract Infection" and the "Respiratory tract infection" and "VVAP"**

We agree that this will be a challenge in the setting of limited diagnostic resources. Hence why we have asked the panel to provide consensus on *suspected, *rather than definite focus, as per the paragraph on page 7 which reads. *The panel will be asked to classify into likely rather than definite focus, because of a desire not to overclassify non-focal sepsis in a setting were lack of diagnostic resources frequently limit the ability to definitively confirm focus of infection.*

**

**P6 – P7: Clinical care and follow-up: it is not clear at which moment the patients will be assessed and by who: if the assessment is done by a trained panel or experts, one can expect sensible and relevant therapeutic and diagnostic actions during the hospital course (e.g. extra cultures at surgical debridement, additional x-ray...). They may influence patient outcome. How will this be handled?**

Patients will be assessed by the study PI (a specialist in infectious diseases) or a clinical member of the study team (clinical officer or junior doctor). Whilst diagnostic and therapeutic recommendations may therefore be made, as this is an observational study, there will be no specific interventions made as part of the study. As each patient is reviewed by a trained clinician, any influence of this on patient outcome will be consistent across the study cohort and should not therefore introduce bias to one patient group over the other. We have clarified this with the following paragraph:

*All participants will be reviewed as soon as possible after recruitment, by the study PI (a specialist in infectious diseases) or a clinical member of the study team and discussed with the PI. If required, recommendations on treatment and diagnostics will be made but as this is an observational study, the care of the patient will be directed by the clinical team responsible for the patient.*

**Table 1: Not clear why Acinetobacter is included – in terms of habitat and behavior, it is different from *Enterobacteriales* as well as from the other Gram-negative fermentative rods.**

During the set-up period for the study, it became clear that 3GC-R *Acinetobacter *spp. were an emerging problem at QECH, particularly amongst neonates. *Acinetobacter*are closely related to *Enterobacterales*, often sharing similar AMR profiles, in particular resistance to ceftriaxone which is the basis of this study.  Given their importance as a nosocomial pathogen, we decided to include these patients in the cohort. However for analysis purposes we may decide to separate *Acinetobacter *from components of the analysis. We have clarified this as a footnote to Table 1.  

**P3: the "counterfactual" approach is not well described for naïve readers – this should be addressed as it is key to the approach.**

We have described this approach in more detail and added additional references:

*The counterfactual assumption is that death would not have occurred if the organism causing the infection had been drug-susceptible (or if there had been no infection). This assumption allows for comparison between patients with resistant and susceptible bacterial infection.*

**

***"Blood cultures which are positive over a weekend will be identified on Monday morning" *meaning that a 2-day delay in identification and antibiotic susceptibility testing will occur and that directed treatment will be delayed – this can be important in situations where empiric treatment did not include effective treatment against Enterobacteriales – is this an issue or is ceftriaxone a part of the standard empiric treatment?**

The blood cultures are reported in real time from the clinical laboratory, including over the weekend, and clinicians can access these results daily. Any weekend delay refers only to recruitment of participants and not routine clinical care. The study team will identify weekend cultures for the purposes of recruitment on a Monday morning, but there would be no expectation of treatment delay. We have adjusted the sentence as follows:

*Blood cultures which become positive over a weekend will be identified by the study **team for recruitment **on a Monday morning.*

**Minor comments**

**Sensitive: suggest to replace by "Susceptible".**

We have made this change. 

**Study Site: for reasons of representativeness, comparison and generalizability, some national demographic and health data could be provided (aside from the GDP).**

We have expanded this paragraph to include further health data.

**Study Site: why are only the admission data for the MJC supplied (and not those for the entire hospital?**

We now include admission data for the hospital.

**Study Site: I suggest to give an overview of the (estimated) distribution adult versus children blood culture samples as well as community versus hospital-associated infections.**

We provide a breakdown of adult versus paediatric blood culture samples. Data on community versus hospital associated infections are currently unknown at QECH. 

**Diagnostic bacteriology: blood volumes sampled are (too) low: this is a reality in low-resource settings but its implications should be discussed – it is possible that mainly patients with high bacterial loads will be selected and included.**

We agree that this is a potential source of bias in the study and although we are limited by space to discuss this in more detail, we have added the following sentence:

*The volume of blood taken for cultures in children in this setting is often low, potentially introducing bias to the study and selecting for patients with high bacterial load. This will be noted as a potential limitation*

**Diagnostic bacteriology: not clear which antibiotics will be tested and of ESBL testing is done.**

We have added an addition table (now Table 2) to describe the AST and a paragraph on ESBL testing.

**Clinical presentation P5: not clear what is meant with " *admission physiology*": vital signs, clinical signs and symptoms, illness severity,...?**

For clarity, we have changed this to admission vital signs. 

**The Abstract might be restructured to give more essential and detailed study information such as the (i) concrete classification of the role of the isolated bacteria and (ii) the healthcare economic data.**

We have restructured the abstract to make it clearer and more detailed. 

**Abstract: Some abbreviations not explained previously (3GC-S) and there is some inconsistency (3GCR-E versus 3GC-R) - probably it will be easier if no abbreviations are used.**

We have provided expansions for 3GC-R, 3GCR-E and 3GC-S.

**In line with the Keywords, "Africa" (L3) might be changed to "sub-Saharan Africa".**

We have made this change. 

**References: Some references are (too) briefly cited (e.g. ref. 15 "World Bank). Suggest to add websites + date of access where appropriate.**

We have made these changes

**Consistency of abbreviations or unusual abbreviation: 3GCR-E versus 3GC-R and DRI – only used a few time in the text.**

We have made these consistent but left them in the text as they are now standard abbreviations in the literature. 

**Style: P4 and elsewhere: paragraph of methods mixes past, future and present tense.**

We have proof read the manuscript to remove any inconsistencies
